# Examining the Branching Patterns of the Hepatis Portae Vena with Computed Tomography Images

**DOI:** 10.3390/jcm14144835

**Published:** 2025-07-08

**Authors:** Bilge Turkmen, Mehmet Tugrul Yilmaz, Duygu Akin Saygin, Cengiz Kadiyoran

**Affiliations:** 1 Healthcare Practice and Research Hospital, Hatay Mustafa Kemal University, Hatay 31100, Türkiye; 2 Department of Anatomy, Faculty of Medicine, Necmettin Erbakan University, Konya 42090, Türkiye; mehmet_tugruly@yahoo.com (M.T.Y.); d.akin.42@yahoo.com (D.A.S.); 3 Department of Radiology, Faculty of Medicine, Necmettin Erbakan University, Konya 42090, Türkiye; ckadiyoran@erbakan.edu.tr

**Keywords:** computed tomography, liver, variation, Hepatis Portae Vena

## Abstract

**Background/Objectives:** The present study aimed to examine the branching pattern images of the Hepatis Portae Vena (HPV), which is one of the vascular structures of the liver, with Computed Tomography (CT), and to uncover the surgical and radiological importance of the variations. **Methods:** The HPV branching patterns on CT images of healthy liver of 996 individuals (47.8% male, 52.2% female) between the ages of 20 and 59 were evaluated according to previously determined definitions. The division of the main branch of the HPV into ramus (r.), dexter, and r. sinister and the later division of r. dexter into r. anterior and r. posterior branches were called Type I-a, other main branch variations were called Type II-a, Type III-a, and Type IV-a, and the r. dexter variations were called Type V-b, Type VI-b, Type VII-b, and Type VIII-b. Also, all individuals in the present study were examined under four age groups as 20–29, 30–39, 40–49, 50–59, and the data were analyzed in the SPSS 21 software. **Results:** Type I-a (73.1%) was detected most frequently in all individuals, but Type VI-b (0.1%) and Type VII-b (0.1%) were detected least frequently. Following Type I-a, Type II-a (10.6%), Type III-a (8.2%), and Type V-b (5.5%) were detected, respectively. No statistically significant differences were detected between gender and age groups in terms of the frequency of HPV types (*p* > 0.05). **Conclusions:** We believe that accurate knowledge and definition of HPV anatomy will guide liver surgeries and interventional radiology, which are the cornerstones of the treatment of liver diseases.

## 1. Introduction

The liver is the largest gland in the body and is an intraperitoneal and parenchymal organ in the upper abdominal cavity [1,2]. As one of the vascular structures of the liver, the HPV is the main blood vessel that carries digestive product-rich, oxygen-poor blood from the gastrointestinal tract (except the lower part of the rectum), spleen, pancreas, and gallbladder to the liver [3,4]. It is a functional vein of the liver and is approximately 8 cm long, formed by the union of the superior mesenteric vein and the splenic vein at the level of L2 vertebra, behind the neck of the pancreatic cortex, contributing to approximately 75–80% of the hepatic blood flow [4,5,6].

In embryological terms, the HPV occurs in the second month of pregnancy by selective involution of the vitelline veins after the anastomoses in front and behind the duodenum. The changes in the obliteration pattern of these anastomoses cause anatomical variations of HPV [7]. In standard HPV anatomy, the main branch of the HPV divides into r. dexter and r. sinister at the liver hilum and r. dexter then gives off the r. anterior and r. posterior branches. Later, r. posterior divides into branches extending to segment VI and segment VII, and r. anterior divides into branches extending to segment V and segment VIII. The r. sinister gives off branches going to segments I, II, III, and IV [3,4].

In the literature, the standard type is defined as Type I in related classifications as the most common type of HPV, and other types are considered as variations [8,9]. HPV variations are common and show regional and geographical differences [7,10,11].

Accurate preoperative assessment of the HPV, which is one of the hepatic vascular structures, other vascular structures, and biliary anatomy, is essential to ensure safe and successful hepatic surgery. Such surgical procedures range from more complex (e.g., partial hepatectomy for tumor resection and living donor liver transplantation) to more routinely performed (e.g., laparoscopic cholecystectomy) procedures [12]. Anatomists and clinicians began to understand the vascularization of the liver in the mid-twentieth century. The studies conducted by Nettelblad in 1954 and Guerrier and Rapp in 1953 laid the foundation for the important study of Claude Couinaud in Paris, France, in 1957 [5]. The study of Couinaud was the first systematic study to examine the arterial, biliary, and venous hepatic vascular patterns of the liver by human cadaveric dissection. Couinaud’s detailed dissections uncovered the classification system, which is known as “the Couinaud Liver Anatomy Classification”, which is used widely by clinicians today [13]. Since then, there have been many studies conducted on the anatomy of the vascular structures of the liver [12,14].

A complete understanding of the standard and variant HPV anatomy and having sufficient knowledge about these variations are critical for radiologists and surgeons to reduce complications and achieve success in surgical interventions in today’s increasing liver transplantation and hepatobiliary interventions [7]. For this reason, the present study aimed to examine the branching patterns of the Hepatis Portae Vena, one of the vascular structures of the liver, on CT images in line with this information and to uncover the surgical importance of the variations.

## 2. Materials and Methods

This study was conducted retrospectively after the approval of the Necmettin Erbakan University Non-Drug and Medical Device Research Ethics Committee (19 January 2024, 2024/4751).

In the present study, 4853 abdomen CT reports obtained with a 64-channel CT device (Somatom Sensation 64, Siemens, Erlangen, Germany) between December 2018 and December 2023 in the archives of the Department of Radiodiagnostics, Faculty of Medicine, Necmettin Erbakan University, were reviewed. The CT images were obtained by connecting a 22-gauge intravenous cannulation to one of the patients’ forearm veins that was visible on the skin and by injecting a total of 100 cc of contrast substance at a rate of 3–4 cc per second in the portal phase (60–65 s after the zero second when the contrast substance was started) according to the determined acquisition parameters (KV: 120, MaS: 86, Effective MaS: 50–170, detector aperture: 1.2 mm, section thickness: 1.5 mm, pitch: 1.4, tube rotation speed: 0.5 s). The images of routine abdomen CT scans were transferred to the workstation in this study (Leonardo Workstation, Siemens Medical Solutions, Erlangen, Germany).

**Figure 1:**
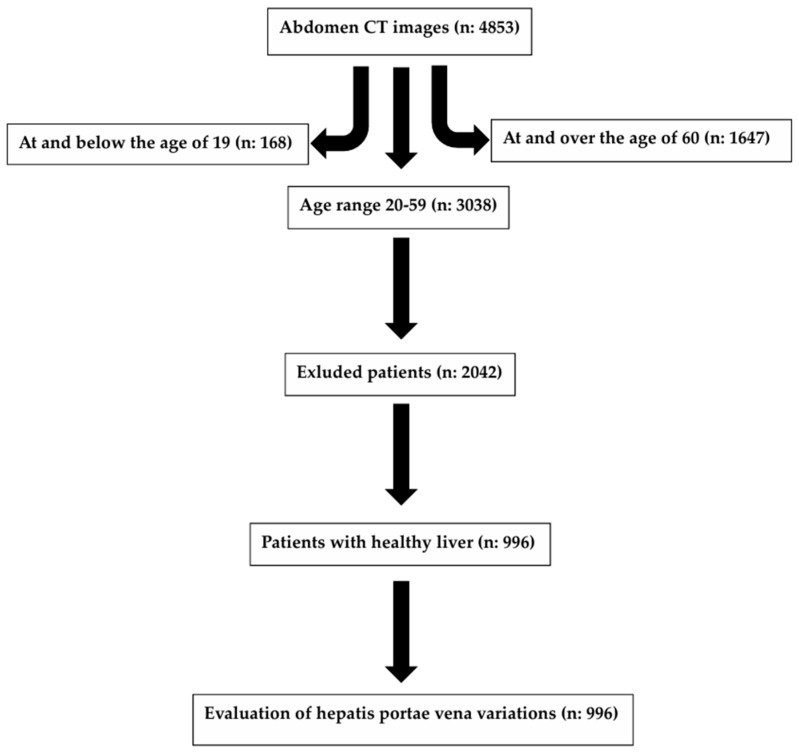
The process of inclusion of the individuals in this study and data collection.

Among the 4853 individuals whose abdominal CT results were reviewed, those younger than 20 years of age (*n*: 168) and older than 59 years of age (*n*: 1647) were not included in this study, and, of the 3038 individuals between the ages of 20 and 59, 2042 individuals who had a history of liver surgery such as hepatic resection, transplantation, etc., focal or multiple lesions affecting liver anatomy, hepatic mass, cirrhosis, hepatomegaly, portal vein embolization, acute or chronic liver disease, a disease affecting the structure of the bile ducts, or a history of gallbladder surgery (biliary obstruction, cholecystectomy, biliary atresia, etc.), and those with inadequate CT image quality were excluded from this study (Figure 1). Individuals from the Central Anatolian population were included in this study to ensure that ethnic/regional differences did not affect the study data. A total of 996 individuals who had healthy livers were included in this study and divided into four different age groups as 20–29 (*n*: 310), 30–39 (*n*: 252), 40–49 (*n:* 243), and 50–59 (*n*: 191). The data on the individuals were obtained by examining the radiological images, evaluation reports, and epicrises. In this study, the evaluation of VPH variations was performed under the supervision of an expert radiologist with 14 years of experience in the field.

### 2.1. Evaluation of Hepatis Portae Vena Variations

**Figure 2:**
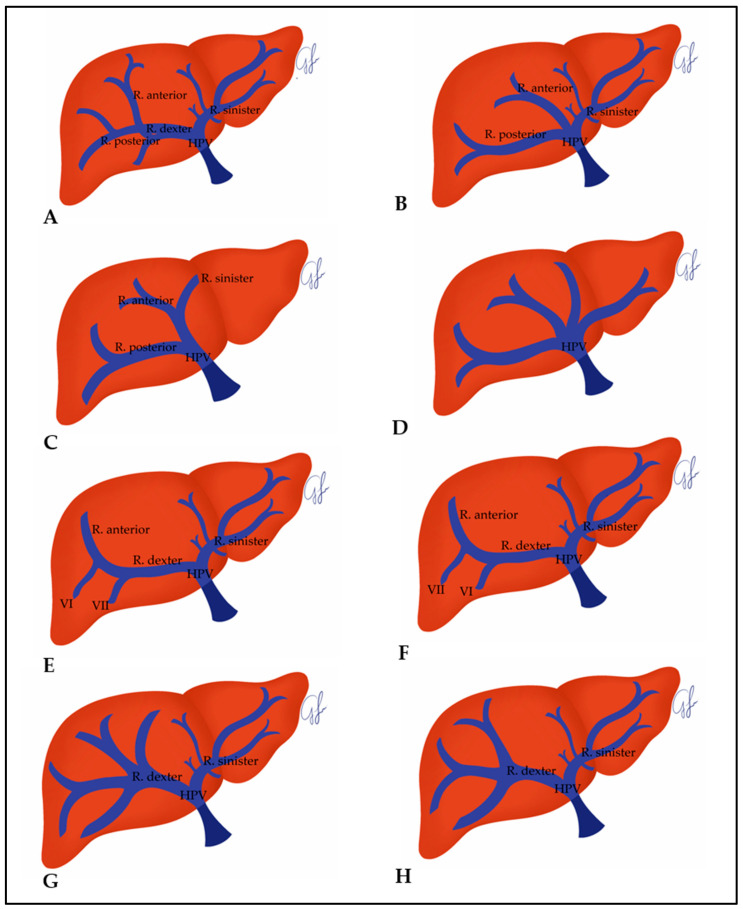
The schematic image of the HPV types in the individuals included in the present study ((**A**) Type I-a HPV, (**B**) Type II-a HPV, (**C**) Type III-a HPV, (**D**) Type IV-a HPV, (**E**) Type V-b HPV, (**F**) Type VI-b HPV, (**G**) Type VII-b HPV, (**H**) Type VIII-b HPV).

The evaluation of HPV variations in 996 individuals with healthy livers was performed based on the definitions in the study of Sureka et al. [15], who used the classification of Covey et al. [16]. The HPV types of the individuals included in the present study were named as Type I-a, Type II-a, Type III-a, Type IV-a, Type Vb, Type VI-b, Type VII-b, and Type VIII-b (Figure 2). Among these, Type Ia, Type II-a, Type III-a, and Type IV-a were main branch variations, Type Vb, Type VI-b, Type VII-b, and Type VIII-b were r. dexter variations.

In the study, the classification made by Covey et al. [16].was used to evaluate the variations of the HPV in 996 individuals with healthy livers. Variations other than this were evaluated according to the definitions made by Sureka et al. [15]. Sureka et al. [15] also divided the variations into groups as VPH main branch variations, r. dexter variations and segmental variations using the classification made by Covey et al. [16] in their study, and created a table defining other variations not defined by Covey et al. [16]. In our study, variations other than the five types determined by Covey et al. [16] were determined and classified according to the table of Sureka et al. [15]. According to this table, Type I-a, Type II-a, Type III-a, and Type IV-a are main branch variations and are added with the suffix “a”, Type V-b, Type VI-b, Type VII-b, and Type VIII-b are ramus dexter variations and are added with the suffix “b”.

Type I-a: The main branch of the HPV divides into r. dexter and r. sinister and r. dexter divides into r. anterior and r. posterior (Figure 2A).

Type II-a: The main branch of the HPV divides into three at the liver hilum as the anterior r., posterior r., and sinister r. (Figure 2B).

Type III-a: The posterior r. first branches off from the main branch of the HPV, and then the anterior r. and r. sinister branch off together as one branch (Figure 2C).

Type IV-a: Main branch of HPV quadrification (Figure 2D).

Type V-b: Segment 7 branch emerges separately from the r. dexter (Figure 2E).

Type VI-b: Segment 6 branch emerges separately from the r. dexter (Figure 2F).

Type VII-b: Quadrification of r. dexter (Figure 2G).

Type VIII-b: Trifurcation of r. dexter (Figure 2H).

### 2.2. Statistical Analysis

The SPSS 21.0 (IBM-Statistics software, Chicago, IL, USA) software was used for the statistical analysis of the data. Descriptive [Mean, Standard Deviation (SD), minimum (min.) and maximum (max.) values] and quantitative analyses [number of individuals (n) and percentages (%)] of the morphological evaluation data were identified. The relationships between the data and gender and descriptive statistics were analyzed by using the Chi-Square Test. The Independent Samples *t*-Test was used to compare the data according to gender, and the One-Way ANOVA (Tukey) Test was used for the evaluations between age groups. The significance level in the evaluations was accepted as *p* < 0.05.

## 3. Results

The data on HPV variations belonged to 996 images (476 male patients, 47.8%; 520 female patients, 52.2%) in the present study. The mean age of the 996 individuals in the 20–59 age group, whose HPV variations were evaluated in the present study, is given in Table 1 based on gender and age groups. According to these data, the mean age of individuals did not show a statistically significant difference according to gender (*p* = 0.331) (Table 1).

The incidence of HPV types according to gender is given in Table 2. Type I-a (73.1%) was detected most frequently in total, and Type VI-b (0.1%) and Type VII-b (0.1%) were detected least frequently. Type II-a (10.7%) was in second place in terms of overall incidence, and Type I-a (73.3%) was detected most frequently in males, and Type IV-a (0.2%), Type VI-b (0.2%), and Type VII-b (0.2%) were detected least frequently. Type III-a (9.7%) was in second place in terms of incidence in males, Type I-a (72.9%) was detected most frequently in females, and Type IV-a (0.2%) was detected least frequently. Type II-a (12.5%) was in second place in terms of incidence in females. Type VI-b and Type VII-b were not detected in females. The incidence of HPV types between genders did not show any statistically significant difference (*p* > 0.05) (Table 2).

The incidence and percentages of HPV types according to age groups are given in Table 3. Type Ia, Type II-a, Type III-a, and Type Vb were detected most frequently in all age groups, respectively. Type VI-b and Type VII-b were not detected in the 20–29 age group, and Type IV-a (0.3%) was least frequently detected. In the 30–39 age group, Type IV-a and Type VI-b were not detected, and Type VII-b (0.4%) was least frequently detected. Type IV-a, Type VI-b, and Type VII-b were not detected in the 40–49 age group, and Type VIII-b (1.6%) was the least frequently detected. While Type VII-b was not detected in the 50–59 age group, Type IV-a (0.5%) and Type VI-b (0.5%) were the least detected. The incidence of HPV types among age groups did not show statistically significant differences (*p* > 0.05) (Table 3).

## 4. Discussion

While some researchers who studied the branching patterns of VPH put forward their own classification methods, others widely used the classifications of researchers such as Atrii et al. [17], Cheng et al. [18], Nakamura et al. [19], and Covey et al. [16], who put forward the first classification methods on the subject in the literature. In the literature, among the classifications, the most common type of HPV is Type I-a, defined as the classical (standard) type; others are considered variations, and Type II-a and Type III-a variations are the most common HPV variations [8,9].

The results of previous studies conducted on HPV variations are given in Table 4. In these studies, it was found that the incidence rate of Type I-a was 51–95.2%, Type II-a was 1.6–29.03%, and Type III-a was 0–23.5%. The findings of the present study were found to be in these ranges, with the incidence rate of Type I-a being 73.1%, Type II-a being 10.7%, and Type III-a being 8.2%.

In the studies given in Table 4, the percentages of other types were Type IV-a 0.1–12%, Type V-a 0.5–3.4%, Type VI-a 1.2–8%, and Type VIII-b 0.9–5.7%. In the present study, Type IV-a was detected at a rate of 0.2%, Type V-b 5.5%, Type VI-b 0.1%, and Type VIII-b 2.1%. The percentage of Type V-b was higher in the present study than in other studies, and the percentage of Type VI-b was lower than those published elsewhere. Type VIII-b was detected only in studies conducted in Türkiye. Type VII-a was detected at a rate of 0.1% in the present study and was detected at a rate of only 4.5% in the study of Liu et al. [7]. The difference in the percentages of HPV branch variations in the studies conducted in our country and on a global scale might have occurred because of the evaluation methods used in these studies, regional and geographical differences, and the number of individuals included. In the literature, HPV variations were evaluated with many different methods such as US (Doppler US), CT (CT angiography, 3D CT, helical CT, etc.), MRI, cholangiography, hepatic arterioportogram, cadaver dissection, and corrosion samples. The most preferred technique for HPV evaluations in these studies was found to be CT, which allows the evaluation of portal blood vessels in high-resolution multiplanar reformations and 3D reconstructions. It is possible to potentially evaluate vascular structures in MRI without employing intravenous contrast and ionizing radiation; however, it is more time-consuming, more sensitive to artifacts, more expensive, and less accessible when compared to CT. Although US also allows venous flow assessment and anatomical information, it has an error rate depending on the person performing it [4]. In previous studies conducted by Tutkuviene et al. [4], it was reported that there were no differences between corrosion casting samples and CT measurement methods in the evaluation of main branch variations in the evaluation of HPV variations, while CT was superior in defining segmental branch variations. It was reported in another study that anatomical variants of HPV showed more variability in cadaveric samples when compared to radiological images, as the dissection allowed greater identification sensitivity [5].

It is extremely important to know HPV variations in surgical procedures such as portal vein embolization, hepatosurgery, liver transplantation surgery, and transjugular intrahepatic portosystemic shunt application [7,15].

Portal vein embolization is an advanced vascular intervention method used to increase the size of the liver and can be performed with the Ipsilateral or Contralateral Approaches. The Ipsilateral Approach is usually preferred to preserve the non-diseased part of the liver. When the anatomy of the HPV is normal, there are few technical difficulties during the Ipsilateral Approach. Complexity emerges when the Contralateral Approach must be used in the Type III-a variation of the HPV. In such cases, a reverse-curved catheter might be required for this procedure. Branching variations of HPV, such as trifurcation and quadrifurcation, which cause difficult and unstable catheterization, cause off-target embolization [15].

As mentioned, HPV variations are extremely important in liver transplantation in terms of creating a reliable hepatectomy plane for donors. In right lobe transplantation, it is important to distinguish between Type II-a and Type III-a variations, and, if Type II-a (trifurcation) is detected, two separate anastomoses might be required [20]. Surgical intervention for donors with Type III-a variation is more complex, and this variant has surgical importance in the donor as well as the recipient [7].

Knowing HPV variations is also extremely important in hepatectomies, especially in right hepatectomies involving segment IV, because embolization of the segment IV branch results in better regeneration of segments I, II, and III. Also, the Type III-a variation is of serious clinical importance as there might be a risk of bleeding from the posterior branch if the surgeon only ligates the right anterior branch [7,15].

It is also very important to know the HPV variations in transjugular intrahepatic portosystemic shunt application, as well as the important anatomical variations are Type II-a and Type III-a variations. An abnormal HPV anatomy might potentially affect transhepatic access [21]. A transjugular intrahepatic portosystemic shunt must be created between the right hepatic vein and the r. dexter of the HPV in most patients. Puncture of the extrahepatic part of the main branch of the HPV during the shunt procedure might cause uncontrolled bleeding [15].

Knowing the HPV variations is also crucial for accurate tumor localization because the branching pattern of the HPV and hepatic veins determines the segmental anatomy of the liver [7].

If there are segmental variations, resection of a particular lobe together with its HPV branch might devascularize a particular segment (especially segments IV and VIII) [15].

Variations in the biliary system morphology are often associated with variations in HPV. Based on the results reported by Lee et al. [22], there is a significant positive association between HPV variants and biliary variants, and patients with HPV variants are more likely to have biliary anomalies, typically involving the hepatic ducts of the right lobe. For this reason, the biliary anatomy of liver transplant donor candidates who have abnormal HPV anatomy must also be carefully analyzed. For example, Yamashita et al. [23] emphasized the relationship between the right-sided round ligament, which is very important for a safe hepatic resection, and structural abnormalities in the biliary and vascular anatomy of the liver. Such variations must be evaluated in detail to minimize the risk of iatrogenic movement because high vascular and biliary variation rates, especially in right lobe liver grafts, are technically more challenging and might cause donor rejection, and are important in liver transplantation because they might cause unintentional bile duct damage [15,22,24].

In the literature, no statistically significant differences were reported between male and female patients in terms of the frequency of HPV variations in the studies of Arviza et al. [5], Koç et al. [8], Kabakcı et al. [6], and Sarı et al. [20]. Similarly, no significant relationships were detected in the present study.

In their study, Kabakcı et al. [6] examined the distribution of HPV types based on age groups and reported that the variations did not show statistically significant differences between age groups. Similarly, in the present study, the frequency and percentages of HPV types did not show statistically significant differences between age groups.

**Table 4:** The comparison of Hepatis Portae Vena variations.

Researchers	Year	Place	Method	*n*	Type I-a (%)	Type II-a (%)	Type III-a (%)	Others (%)
Atrii [17]	1992	Canada	US	507 patients	80	10.8	4.7	4.5
Soyer et al. [25]	1994	France	Helical CT during arterial US	69 patients	94	4	-	2
Cheng et al. [18]	1997	China	Cholangiography and hepatic arterioportogram	210 patients	69.52	19.05	4.29	7.14
Baba et al. [26]	2000	Japan	Helical CT with arterial portography	192 healthy	89.1	5.2	2.6	5.7
Nakamura et al. [19]	2002	Japan	CT and Doppler US	120 graft donors	92.5	2.5	2.5	2.5
Akgul et al. [13]	2002	Turkiye	CT	585	86.2	12.3	1.5	-
Covey et al. [16]	2004	New York	CT portography	200 healthy	65	9	13	13
Lee et al. [22]	2004	New York	MRI	108	89	4	-	7
Atasoy and Ozyurek [27]	2005	Turkiye	CT	200	65.5	9.5	23.5	1.5
Koc et al. [8]	2008	Turkiye	CT	1384 healthy	75.5	11.1	9.7	3.7
Okten et al. [28]	2010	Turkiye	CT	85 donors	65.88	10.58	22.35	1.17
Yaprak et al. [29]	2011	Turkiye	Volumetric CT, CT angiography, MRI cholangiography	200 healthy living donors	85	4.5	5	5.5
Munguti et al. [30]	2012	Kenya	Dissection	100	51	22	15	12
Guler et al. [31]	2013	Turkiye	CT	386 healthy liver donors	86.5	5.18	6.21	2.07
Sari et al. [20]	2012	Turkiye	CT	48 donors	52	14.6	8.4	25
Takeishi et al. [32]	2014	Japan	CT cholangiography	407	89	6.1	4.7	0.4
Sureka et al. [15]	2015	New Delhi	CT	967 healthy	79.94	6.83	4.96	8.27
Watanabe et al. [10]	2016	Japan	CT	200	86.0	4.5	9.5	-
			Surgical method	463	86.3	4.8	8.9	-
Gunasekaran et al. [21]	2017	USA	CT, MRI, angiography	100	67	10	6	17
Yanmaz and Karazincir [33]	2017	Turkiye	CT	750 patients	82.1	9.6	7.1	1,2
Ulger et al. [14]	2017	Turkiye	CT, angiography, and MRI cholangiopancreatography	200 healthy	76	9	8.5	6.5
Kuriyama et al. [34]	2018	Japan	CT	149 donors	83.5	4.7	11.4	-
Minami et al. [35]	2019	Japan	CT	100 healthy	87	5	8	-
Clipet et al. [11]	2019	France	CT	346	71	17	-	12
Anwar et al. [36]	2020	England	CT angiography	500 healthy	95.2	1.6	2.4	0.8
Asad Ullah et al. [24]	2020	Pakistan	CT	500 healthy	87.6	3.6	4.4	4.4
Ulusoy et al. [37]	2020	Turkiye	CT	838 healthy	82.6	8.6	-	8.8
Kabakci et al. [6]	2020	Turkiye	CT	340 healthy	83.82	10	4.11	2.05
Arviza et al. [5]	2021	Spain/Austria	Cadaver dissection	31	61.2	29.03	-	9.67
			CT	216	66.66	12.03	14,35	6.94
Tutkuviene et al. [4]	2024	Lithuanian	Corrosion sample and CT	105 corrosion samples	85.7	7.6	7	1
Karakaya et al. [38]	2024	Turkiye	CT	287 healthy donor candidates	76.3	13.9	9.8	-
Liu et al. [7]	2024	Chinese	CT	178 healthy	62.9	12.9	3.9	20.3
Our study	2024	Turkiye	CT	996 healthy	73.1	10.7	8.2	8

(n: number of individuals, % percentage, CT: Computed Tomography, US: Ultrasonography, MRI: Magnetic Resonance Imaging).

## 5. Conclusions

In recent years, significant advances in the understanding of the vascular and functional anatomy of the liver, alongside innovations in surgical techniques, such as navigation-guided and robotic-assisted procedures, have profoundly influenced the current landscape of liver surgery. However, hepatic surgery, which is an essential component in the management of various liver diseases, continues to carry considerable morbidity risks, largely due to the complex and variable nature of liver anatomy.

In this context, our study offers valuable insights by characterizing the HPV morphology in a large cohort of 996 healthy individuals. To our knowledge, this constitutes one of the most comprehensive single-center datasets on portal vein anatomical variations within a Central Anatolian population. Unlike previous studies, our research emphasizes the clinical relevance of these anatomical differences, each of which may bear critical implications for specific surgical procedures.

Accurate delineation of HPV anatomy is essential, as specific variants may influence key intraoperative decisions, including vascular control, parenchymal transection, and anastomosis planning. The integration of advanced 3D imaging and modeling techniques into preoperative workflows is strongly advocated, as it facilitates precise anatomical visualization, thereby minimizing the risk of complications such as non-target embolization, donor-recipient mismatch, and iatrogenic bile duct injury. Our dataset not only enriches the anatomical literature but also supports the refinement of surgical planning protocols through patient-specific vascular mapping.

## Figures and Tables

**Table 1:** The mean age of the individuals whose Vena Portae Hepatis variations were evaluated according to gender and age groups.

		*n*	Mean ± SD
**Gender**	Male	476	37.64 ± 11.4
	Female	520	37.31 ± 11.35
**Age group**	20–29 age group	310	24.21 ± 2.82
	30–39 age group	252	34.71 ± 2.92
	40–49 age group	243	44.14 ± 2.93
	50–59 age group	191	54.15 ± 2.87

(*n*: number of individuals, Mean ± SD: Mean ± Standard Deviation).

**Table 2:** The prevalence of Hepatis Portae Vena types according to gender.

	Total *n* (%)	Male *n* (%)	Female *n* (%)	χ^2^	*p*
Type I-a	728 (73.1)	349 (73.3)	379 (72.9)	9.318	0.231
Type II-a	106 (10.7)	41 (8.6)	65 (12.5)		
Type III-a	82 (8.2)	46 (9.7)	36 (6.9)		
Type IV-a	2 (0.2)	1 (0.2)	1 (0.2)		
Type V-b	55 (5.5)	29 (6.1)	26 (5.0)		
Type VI-b	1 (0.1)	1 (0.2)	-		
Type VII-b	1 (0.1)	1 (0.2)	-		
Type VIII-b	21 (2.1)	8 (1.7)	13 (2.5)		
Total	996 (100.0)	476 (100.0)	520 (100.0)		

(***n****:* number of individuals, %: percentage, χ^2^: Chi-Square Test, *p* < 0.05 significance).

**Table 3:** The incidence of Hepatis Portae Vena types according to age groups.

	20–29 Age Group *n* (%)	30–39 Age Group *n* (%)	40–49 Age Group *n* (%)	50–59 Age Group *n* (%)	χ^2^	*p*
Type I-a	226 (72.9)	183 (72.6)	193 (79.4)	126 (66.0)	22,008	0.399
Type II-a	33 (10.7)	25 (9.9)	19 (7.9)	29 (15.2)		
Type III-a	28 (9.0)	20 (7.9)	17 (7.0)	17 (8.9)		
Type IV-a	1 (0.3)	-	-	1 (0.5)		
Type V-b	17 (5.5)	15 (6.0)	10 (4.1)	13 (6.8)		
Type VI-b	-	-	-	1 (0.5)		
Type VII-b	-	1 (0.4)	-	-		
Type VIII-b	5 (1.6)	8 (3.2)	4 (1.6)	4 (2.1)		
Total	310 (100)	252 (100)	243 (100)	191 (100)		

(***n*:** number of individuals, %: percentage, AR: Age Range, χ^2^: Chi-Square Test, *p* < 0.05 significance).

## Data Availability

The data that support the findings of this study are not publicly available due to ethical restrictions, and they are available from the corresponding author upon reasonable request.

## Abbreviations

HPV	Hepatis Portae Vena
CT	computed tomography
R	ramus
US	ultrasonography
MRI	magnetic resonance imaging
